# Depletion of Extracellular Chemokines by Aspergillus Melanin

**DOI:** 10.1128/mbio.00194-23

**Published:** 2023-04-17

**Authors:** Karen T. Graf, Hong Liu, Scott G. Filler, Vincent M. Bruno

**Affiliations:** a Institute for Genome Sciences, University of Maryland School of Medicine, Baltimore, Maryland, USA; b Division of Infectious Diseases, Lundquist Institute for Biomedical Innovation at Harbor-UCLA Medical Center, Torrance, California, USA; c David Geffen School of Medicine at UCLA, Torrance, California, USA; d Department of Microbiology and Immunology, University of Maryland School of Medicine, Baltimore, Maryland, USA; IMBB-FORTH

**Keywords:** *Aspergillus fumigatus*, airway epithelial cells, chemokines, CXCL10, CCL20, melanin

## Abstract

Aspergillus fumigatus is an environmental fungus that can cause life-threatening pulmonary disease. Infections initiate when conidia are inhaled and land deep inside the small airways and alveoli of the lungs, where they interact with epithelial cells. These cells provide a physical barrier and secrete chemokines to attract innate immune cells to the site of infection. Melanin, a key constituent of the conidial cell wall, is required for the establishment of invasive infection due to its ability to inhibit the function of innate immune cells recruited to clear the infection. Here, we provide evidence for an additional mechanism by which A. fumigatus can alter host innate immune responses. *In vitro* infection of a normal human small airway epithelial cell line (HSAEC1-KT) caused a decrease in extracellular protein levels of CXCL10 and CCL20, two proinflammatory chemokines that are required for the host defense against aspergillosis, despite a dramatic increase in the levels of each mRNA. A. fumigatus depleted recombinant human CXCL10 and CCL20 from medium in the absence of host cells, suggesting that the block in accumulation is downstream of protein translation and secretion. Melanin is both necessary and sufficient for this chemokine-depleting activity because a dihydroxynaphthalene (DHN)-melanin-deficient strain of A. fumigatus is defective in depleting chemokines and purified melanin ghosts retain potent depletion activity. We propose that A. fumigatus, through the action of melanin, depletes important chemokines, thereby dampening the innate immune response to promote infection.

## OBSERVATION

**FIG 1:**
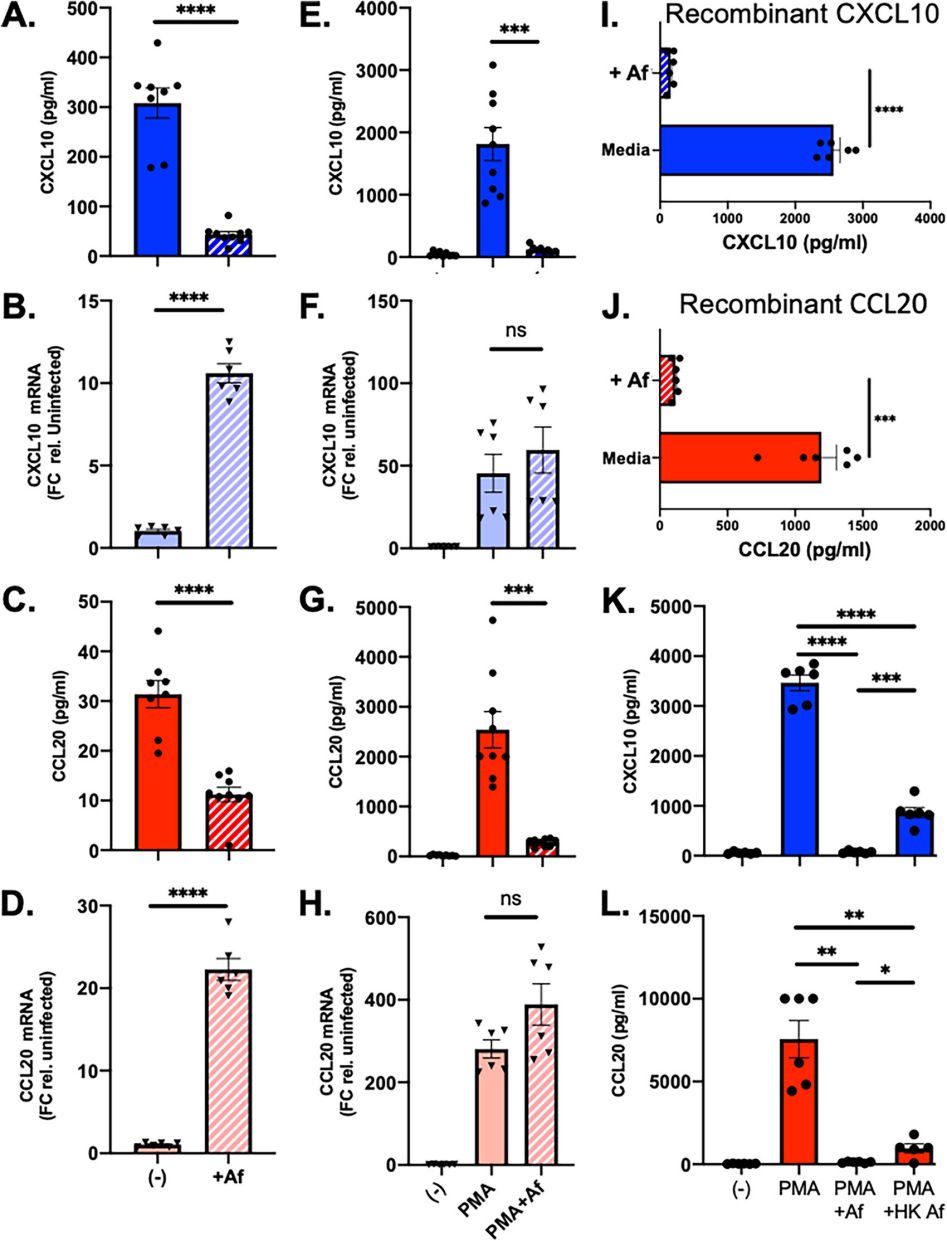
A. fumigatus can deplete CXCL10 and CCL20 from culture supernatants of airway epithelial cells. (A to D) HSAEC1-KT cells were infected with A. fumigatus conidia (strain Af293) for 24 h, after which the levels of CXLC10 and CCL20 protein levels were determined in the culture supernatants by ELISA (A and C) and the mRNA levels for each gene were determined by quantitative reverse transcription-PCR (qRT-PCR) (B and D). (E to H) HSAEC1-KT cells were treated with PMA in the presence or absence of A. fumigatus conidia (Af293) for 6 h, after which the levels of CXLC10 and CCL20 protein levels were determined in the culture supernatants by enzyme-linked immunosorbent assay (ELISA) (E and G) and the mRNA levels for each gene were determined by qRT-PCR (F and H). (I and J) Recombinant human CXCL10 (I) or CCL20 (J) was added to tissue culture medium and incubated in the presence or absence of A. fumigatus conidia (Af293). Protein levels were measured by ELISA following 6 h of incubation. (K and L) The experiment from panels E and G was repeated with either live or heat-killed conidia of isolate Af293. Values represent the mean ± standard error of the mean (SEM) from at least 2 experiments, each performed in triplicate. ****, *P* < 0.0001; ***, *P* < 0.001; **, *P* < 0.01; *, *P* < 0.05; ns, not significant; HK, heat killed; Af, A. fumigatus; (−), untreated control. Detailed methods are described in Text S1 in the supplemental material.

Invasive pulmonary aspergillosis is initiated when Aspergillus fumigatus conidia adhere to and invade lung epithelial cells. In addition to forming a physical barrier, these cells are immunologically active and contribute to host defense through the secretion of chemokines, which recruit innate immune cells to the site of infection (1). As part of our initial characterization of the interaction between A. fumigatus and a Tert-immortalized human small airway epithelial cell line (HSAEC1-KT), we measured the secreted protein levels of interleukin-1α (IL-1α), C-X-C motif ligand 8 (CXCL8), CXCL10, and C-C motif chemokine ligand 20 (CCL20) following *in vitro* infection with the Af293 strain. These proteins are known to be secreted by airway epithelial cells in response to microbial infection (1–4) and are important in the host defense against pulmonary aspergillosis (5–11). While the levels of both mRNA and secreted protein for IL-1α and CXCL8 increased in response to infection (see Fig. S1 in the supplemental material), the levels of CXCL10 and CCL20 in the culture supernatant decreased (Fig. 1A and C). This decrease in secreted protein levels occurred despite a very strong upregulation of both mRNAs in response to infection (Fig. 1B and D). We also observed a discordance between mRNA expression and extracellular protein secretion for both chemokines following *in vitro* infection with a different A. fumigatus isolate, CEA10 (Fig. S2). These data suggest that A. fumigatus can inhibit the accumulation of CXCL10 and CCL20 in culture supernatants in a manner that is independent of the transcriptional regulation of both genes.

10.1128/mbio.00194-23.2 FIG S1 Quantitation of IL-1α and CXCL8 mRNA and secreted protein levels following infection of HSAEC1-KT cells with A. fumigatus strain Af293. HSAEC1-KT cells were infected with A. fumigatus conidia (strain Af293) for 24 h, after which the IL-1α and CXCL8 secreted protein levels were determined in the culture supernatants by ELISA (A and C) and mRNA levels were measured by qRT-PCR (B and D). Values represent the mean ± SEM from at least 2 experiments, each performed in triplicate. ****, *P* < 0.0001; ***, *P* < 0.001. Download FIG S1, PDF file, 0.1 MB. Copyright © 2023 Graf et al. 2023 Graf et al. https://creativecommons.org/licenses/by/4.0/ This content is distributed under the terms of the Creative Commons Attribution 4.0 International license.

10.1128/mbio.00194-23.3 FIG S2 Quantitation of CXCL10 and CCL20 mRNA and secreted protein levels following infection of HSAEC1-KT cells with A. fumigatus strain CEA10. HSAEC1-KT cells were infected with A. fumigatus conidia (strain CEA10) for 24 h, after which the levels of CXLC10 and CCL20 protein levels were determined in the culture supernatants by ELISA (A and C) and the mRNA levels for each gene were determined by qRT-PCR (B and D). Values represent the mean ± SEM from 1 to 2 experiments, each performed in triplicate. ****, *P* < 0.0001; ***, *P* < 0.001; **, *P* < 0.01; ns, not significant. Download FIG S2, PDF file, 0.09 MB. Copyright © 2023 Graf et al. 2023 Graf et al. https://creativecommons.org/licenses/by/4.0/ This content is distributed under the terms of the Creative Commons Attribution 4.0 International license.

10.1128/mbio.00194-23.1 TEXT S1 Detailed descriptions of the materials and methods used in this work. Download Text S1, DOCX file, 0.05 MB. Copyright © 2023 Graf et al. 2023 Graf et al. https://creativecommons.org/licenses/by/4.0/ This content is distributed under the terms of the Creative Commons Attribution 4.0 International license.

**FIG 2:**
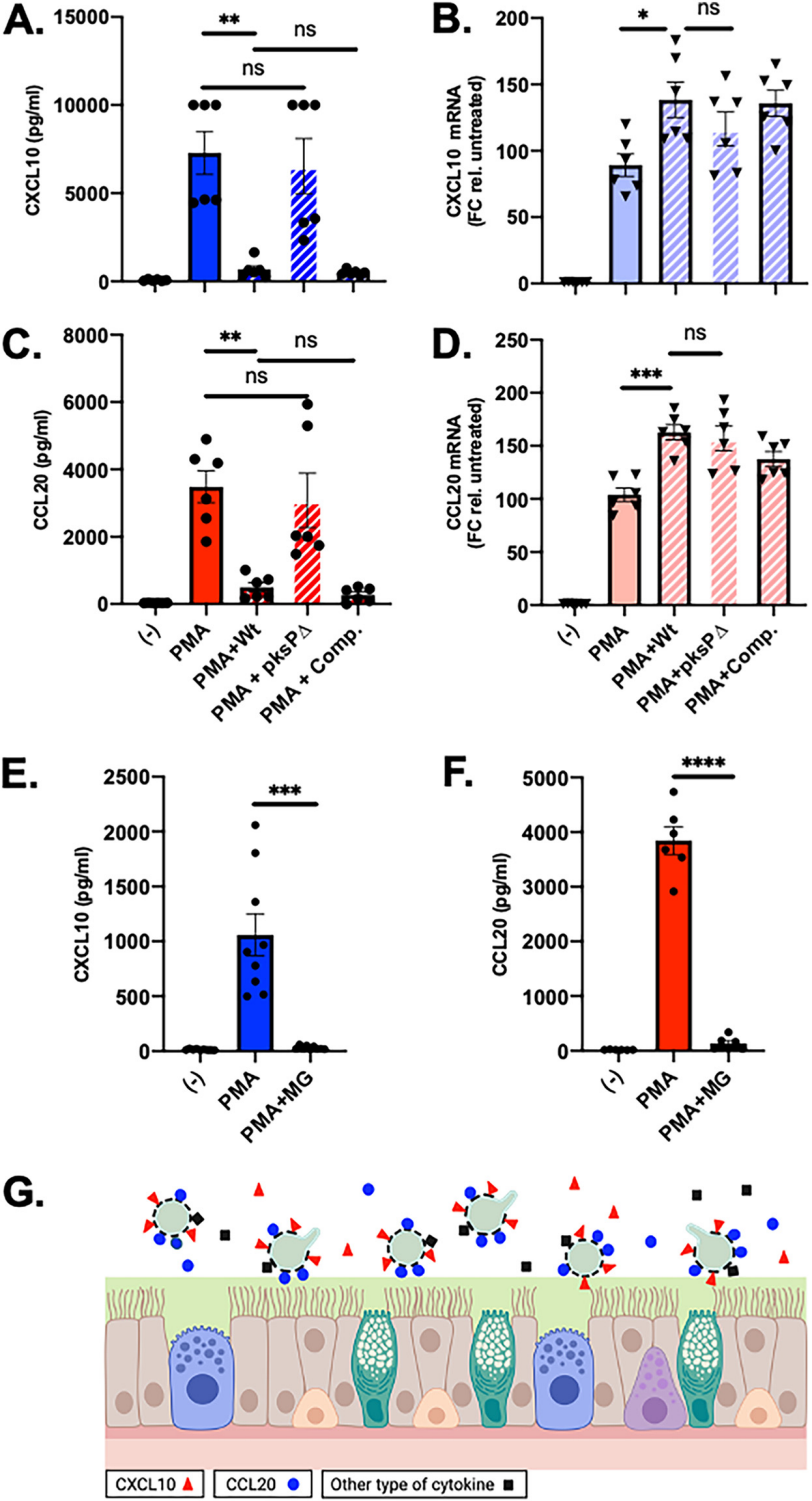
A. fumigatus melanin is necessary and sufficient for depletion of CXCL10 and CCL20. (A to D) HSAEC1-KT cells were treated with PMA in the presence or absence of the designated A. fumigatus conidia (from the B5233 isolate background) for 6 h, after which the levels of CXLC10 and CCL20 protein levels were determined in the culture supernatants by ELISA (A and C) and the mRNA levels for each gene were determined by qRT-PCR (B and D). (E and F) The experiment from panels A and C was repeated with the addition of purified melanin ghosts from A. fumigatus (Af293) with a multiplicity of 3 melanin ghosts per host cell. (G) Schematic of our proposed model by which A. fumigatus dampens innate immunity by depleting CXCL10, CCL20, and potentially other cytokines from the extracellular space. Values represent the mean ± SEM from at least 2 experiments, each performed in triplicate. ****, *P* < 0.0001; ***, *P* < 0.001; **, *P* < 0.01; *, *P* < 0.05; ns, not significant; (−), untreated control; MG, melanin ghosts. Detailed methods are described in Text S1 in the supplemental material.

We next tested the ability of A. fumigatus to prevent the accumulation of CXCL10 and CCL20 protein when produced in response to an independent stimulus. Phorbol esters, including phorbol 12-myristate 13-acetate (PMA), are potent activators of protein kinase C (PKC) signaling and induce profound changes in gene expression in many different cell types (12–14). Exposure of HSAEC1-KT cells to PMA resulted in a robust induction of both CXCL10 and CCL20 mRNAs (Fig. 1F and H) and secreted CXCL10 and CCL20 protein levels (Fig. 1E and G). Addition of A. fumigatus (Af293) conidia to the cultures of PMA-treated HSAEC1-KT cells almost completely abolished the accumulation of secreted CXCL10 and CCL20 in the culture supernatant without reducing the PMA-induced expression of either mRNA (Fig. 1). We obtained similar results with two additional A. fumigatus isolates, CEA10 and B5233 (Fig. 2A to D; Fig. S3).

10.1128/mbio.00194-23.4 FIG S3 Quantitation of CXCL10 and CCL20 mRNA and secreted protein levels following treatment of HSAEC1-KT cells with PMA in the presence or absence of A. fumigatus strain CEA10. HSAEC1-KT cells were treated with PMA in the presence or absence of A. fumigatus conidia (CEA10) for 6 h, after which the CXLC10 and CCL20 protein levels were determined in the culture supernatants by ELISA (A and C) and the mRNA levels for each gene were determined by qRT-PCR (B and D). Values represent the mean ± SEM from 1 to 2 experiments, each performed in triplicate. ****, *P* < 0.0001; ***, *P* < 0.001; *, *P* < 0.05. Download FIG S3, PDF file, 0.09 MB. Copyright © 2023 Graf et al. 2023 Graf et al. https://creativecommons.org/licenses/by/4.0/ This content is distributed under the terms of the Creative Commons Attribution 4.0 International license.

We wondered if the block in extracellular accumulation of CXCL10 and CCL20 was the result of (i) fungal inhibition of translation, (ii) fungal inhibition of protein secretion, or (iii) the removal of secreted chemokines from the medium. We reasoned that if the block in accumulation occurred downstream of protein production and secretion, A. fumigatus would be able to deplete CXCL10 and CCL20 from medium in the absence of HSAEC1-KT cells. To address this, we tested the ability of A. fumigatus (Af293) to remove recombinant human CXCL10 and CCL20 from medium in the absence of HSEAC1-KT cells. Incubation of recombinant human CXLC10 or CCL20 with A. fumigatus reduced the amount of recombinant protein by greater than 90% (Fig. 1I and J). Notably, incubation of A. fumigatus (Af293) with recombinant human IL-1α or CXCL8 did not reduce the amount of either protein from the medium (Fig. S4). While we have not experimentally ruled out inhibition of translation or protein secretion by A. fumigatus, these results are consistent with a model in which secreted CXCL10 and CCL20 are removed from the medium by A. fumigatus. Importantly, the removal of CXCL10 and CCL20 from the medium may only be responsible for part of the depletion that we observe.

10.1128/mbio.00194-23.5 FIG S4 Quantitation of recombinant IL-1α and CXCL8 protein levels following incubation with conidia of A. fumigatus strain Af293 in the absence of host cells. Recombinant human IL-1 or human CXCL8 was added to tissue culture medium and incubated in the presence or absence of A. fumigatus conidia (Af293). Protein levels were measured by ELISA following 6 h of incubation. ns, not significant. Download FIG S4, PDF file, 0.06 MB. Copyright © 2023 Graf et al. 2023 Graf et al. https://creativecommons.org/licenses/by/4.0/ This content is distributed under the terms of the Creative Commons Attribution 4.0 International license.

In order to understand the mechanism by which A. fumigatus depletes CXCL10 and CCL20 from the culture supernatants, we performed the PMA induction assay with modifications. The presence of a protease inhibitor cocktail in the tissue culture medium had a very modest effect on chemokine accumulation (Fig. S5), and heat-killed conidia were able to significantly deplete CXCL10 and CCL20, although the chemokine levels were slightly higher in these samples than in live conidia (Fig. 1K and L). These results suggest A. fumigatus conidia do not need to be alive in order to exert this cytokine-depleting activity and the activity in not likely to be a result of degradation by a fungal protease. The fungal molecule responsible for this activity is likely to be heat stable.

10.1128/mbio.00194-23.6 FIG S5 Quantitation of CXCL10 and CCL20 secreted protein levels following treatment with PMA and A. fumigatus strain Af293 in the presence of a protease inhibitor cocktail. (A and B) HSAEC1-KT cells were treated with PMA in the presence or absence of A. fumigatus conidia (Af293) and the presence or absence of a protease inhibitor cocktail for 6 h, after which the CXLC10 (A) and CCL20 (B) protein levels were determined in the culture supernatants by ELISA. Values represent mean ± SEM. Download FIG S5, PDF file, 0.04 MB. Copyright © 2023 Graf et al. 2023 Graf et al. https://creativecommons.org/licenses/by/4.0/ This content is distributed under the terms of the Creative Commons Attribution 4.0 International license.

We next conducted a set of experiments to explore the specific role of melanin in the chemokine-depleting activity. The most abundant form of melanin in A. fumigatus is dihydroxynaphthalene (DHN)-melanin, which is synthesized by a biosynthetic cluster of proteins encoded by *pksP*, *ayg1*, *arp2*, *arp1*, and *abr2*. The *pksP* gene encodes a polyketide synthase, which catalyzes the first step in the biosynthetic pathway (15). Strains carrying loss-of-function mutations in *pksP* produce white conidia that lack melanin (16–18). In our PMA induction assay, we chose to test the *pksP* deletion (*pksp*Δ) strain generated by Tsai et al. alongside the parental wild-type strain (B5233) and a complemented strain in which *pksP* activity was restored (18). Both the wild-type strain and the complemented strain reduced the amount of PMA-induced accumulation of CXCL10 and CCL20 by over 90% (Fig. 2A and C). In contrast, the *pksP*Δ strain showed no difference compared to the PMA induction in the absence of A. fumigatus (Fig. 2A and C). The differences in protein accumulation between the strains in these experiments were not a result of A. fumigatus reducing the PMA-induced mRNA levels (Fig. 2B and D). A. fumigatus melanin appears to be sufficient for this chemokine-depleting activity since addition of purified A. fumigatus melanin particles (melanin ghosts) alone abolished PMA-induced accumulation of both CXCL10 and CCL20 (Fig. 2E and F). The ability of melanin ghosts to deplete the chemokines was dose dependent (Fig. S6). Taken together, these results provide direct evidence that A. fumigatus melanin can deplete extracellular CXCL10 and CCL20.

10.1128/mbio.00194-23.7 FIG S6 Quantitation of CXCL10 and CCL20 secreted protein levels following treatment of HSAEC1-KT cells with PMA in the presence or absence of melanin ghosts derived from A. fumigatus strain Af293. HSAEC1-KT cells were treated with PMA in the presence or absence of live Af293 spores (multiplicity of infection [MOI] of 5) or melanin ghosts purified from A. fumigatus (Af293) for 6 h, after which the CXLC10 (A) and CCL20 (B) protein levels were determined in the culture supernatants by ELISA. The number of melanin ghosts added ranged from 5 to 0.1 per host cell. Values represent the mean ± SEM. **, *P* < 0.01; *, *P* < 0.05. Download FIG S6, PDF file, 0.06 MB. Copyright © 2023 Graf et al. 2023 Graf et al. https://creativecommons.org/licenses/by/4.0/ This content is distributed under the terms of the Creative Commons Attribution 4.0 International license.

### Conclusions.

Based on our observations, we propose a novel mechanism by which A. fumigatus melanin suppresses host innate immunity to promote infection by binding to and removing CXCL10 and CCL20 from the extracellular environment (Fig. 2G). The importance of CXCL10 and CCL20 in the host defense against A. fumigatus is supported by published findings from other groups. First, allogeneic stem cell transplant patients carrying single nucleotide polymorphisms (SNPs) in CXCL10 are hypersusceptible to developing invasive aspergillosis (7, 11). Second, mice carrying a deletion of CXCR3, the receptor for CXCL10, have decreased survival and increased fungal burden following challenge with A. fumigatus compared to wild-type controls (8). Third, mice carrying a deletion in the CCR6 gene, which encodes the receptor for CCL20, are also hypersusceptible to A. fumigatus infection (19).

Proteins have been found to be intimately associated with melanin granules from Cryptococcus neoformans (20), providing a precedent for a potential interaction between a host protein and fungal melanin. Also, A. fumigatus strains that lack the ability to produce DHN-melanin have attenuated virulence (16–18, 21). In addition to conferring resistance to reactive oxygen species (ROS), melanin from A. fumigatus can regulate host immune responses by sequestering calcium to prevent calmodulin recruitment to the phagolysosome and subsequent LC3-associated phagocytosis (LAP) (22, 23). Furthermore, a *pksP*Δ mutant induces higher levels of *in vitro* transepithelial neutrophil migration than a wild-type strain, suggesting that DHN-melanin can inhibit this process (24). Our results provide an additional mechanism by which melanin contributes to innate immune regulation by A. fumigatus (Fig. 2G). At the moment, the molecular basis by which CXCL10 and CCL20 are depleted by Aspergillus melanin remains unknown and elucidation of these mechanisms requires additional experiments. Further experiments are also required to determine the complete array of secreted host proteins that can be depleted by A. fumigatus as well as the immunological consequences of CXCL10 and CCL20 depletion during A. fumigatus infection.
