# Supplementary figures and images for: Depletion of Extracellular Chemokines by Aspergillus Melanin
Source: mBio. 2023 Apr 17;14(3):e00194-23. doi: 10.1128/mbio.00194-23 (PMC10294650; doi:10.1128/mbio.00194-23)

Figure S1

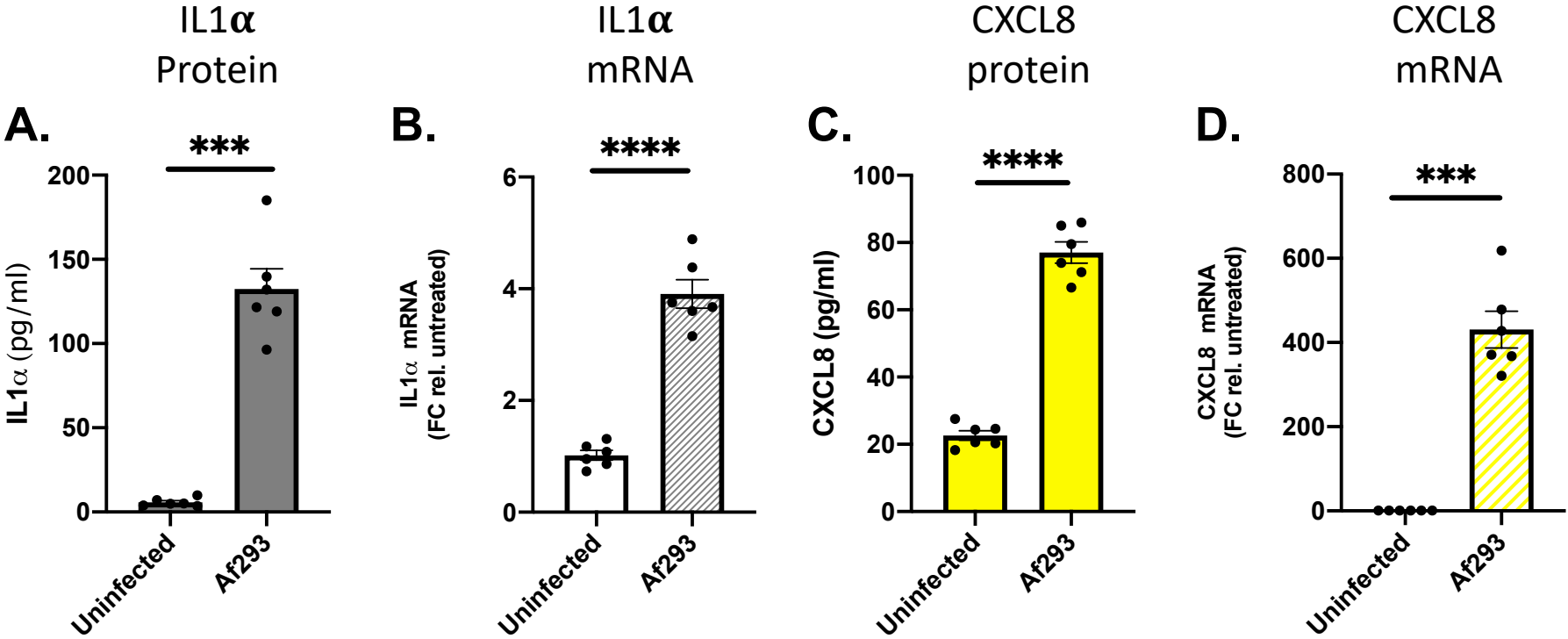

Supplement: FIG S1 [file mbio.00194-23-s0002.pdf]

Figure S2

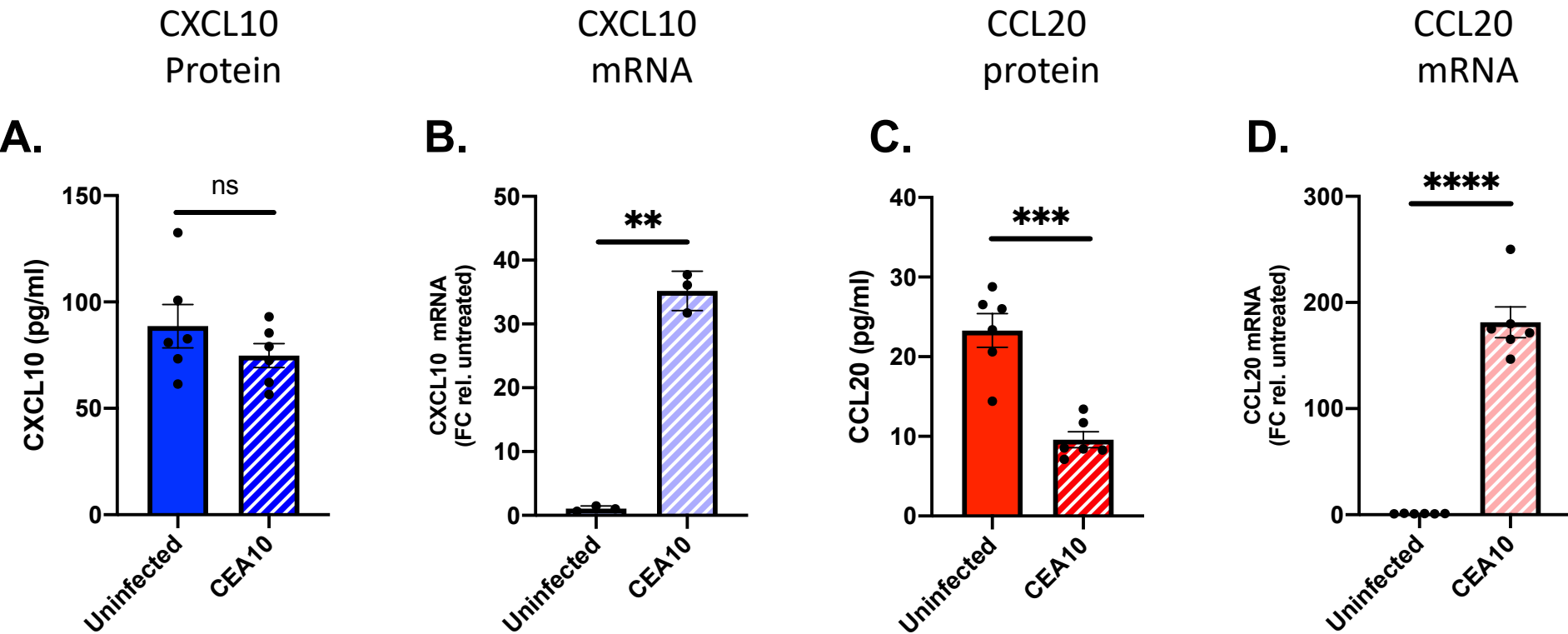

Supplement: FIG S2 [file mbio.00194-23-s0003.pdf]

Figure S3

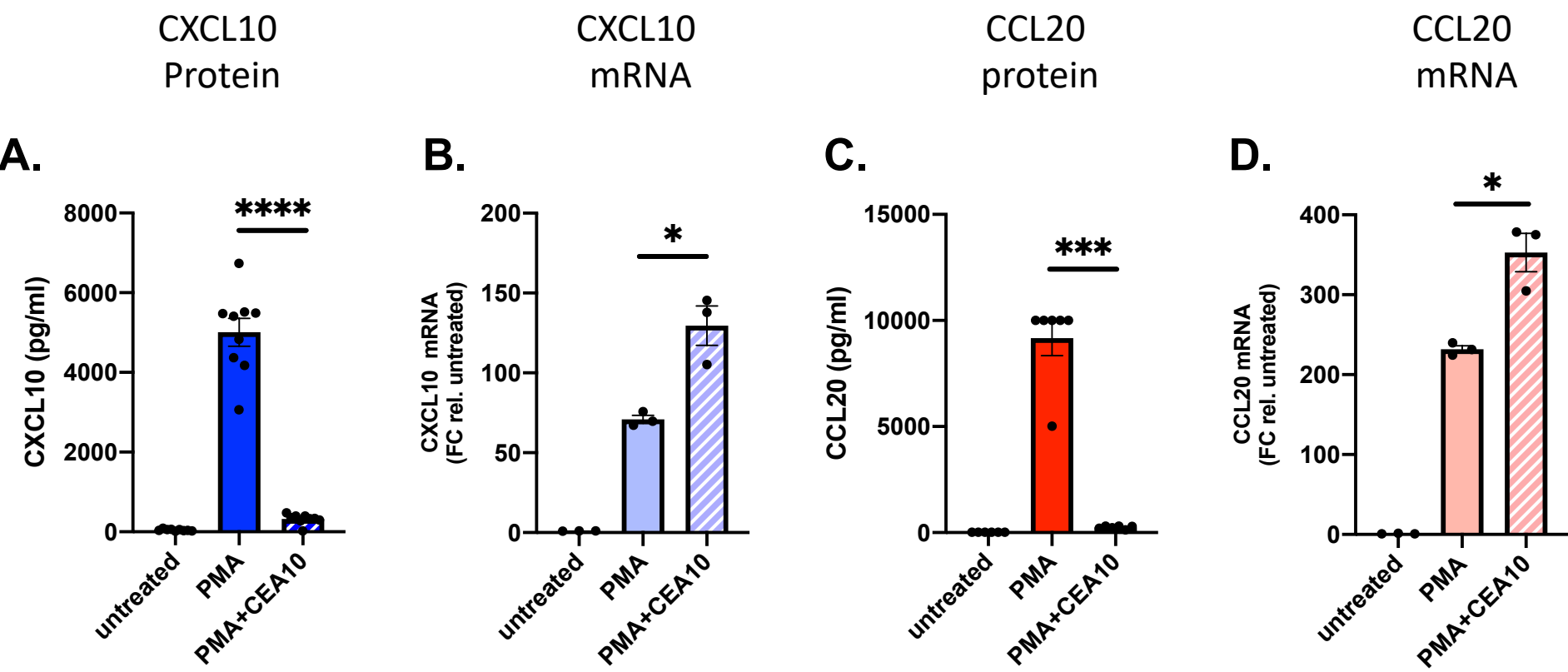

Supplement: FIG S3 [file mbio.00194-23-s0004.pdf]

Figure S4

Recombinant IL1 $\alpha$

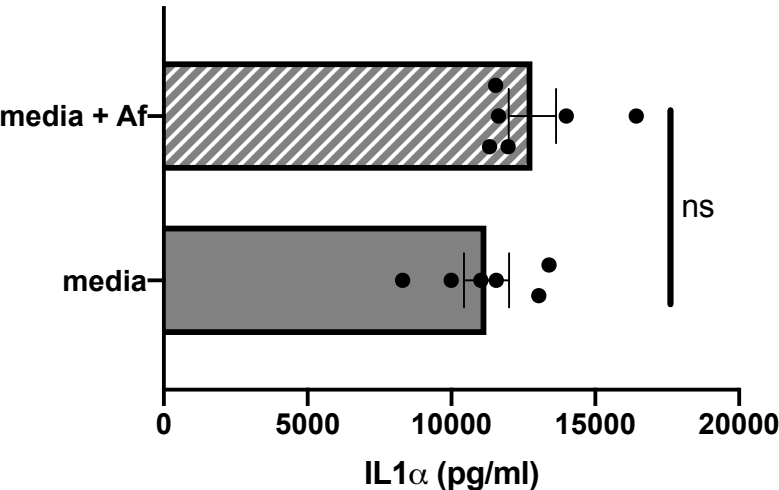

Recombinant CXCL8

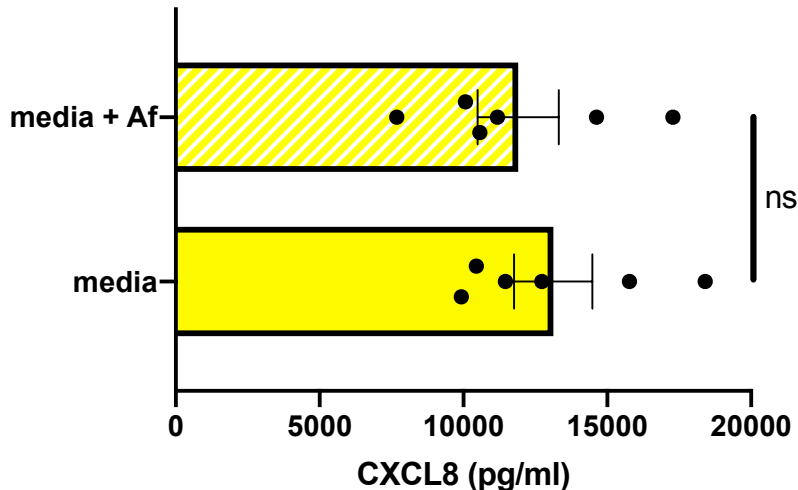

Supplement: FIG S4 [file mbio.00194-23-s0005.pdf]

Figure S5

**A.**

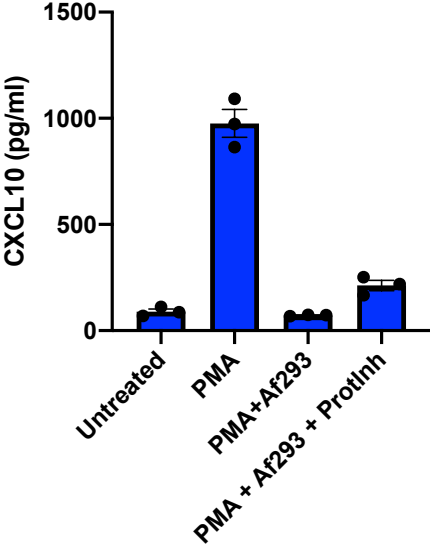

**B.**

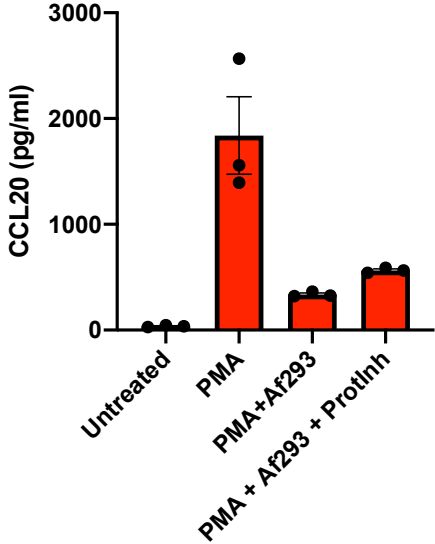

Supplement: FIG S5 [file mbio.00194-23-s0006.pdf]

Figure S6

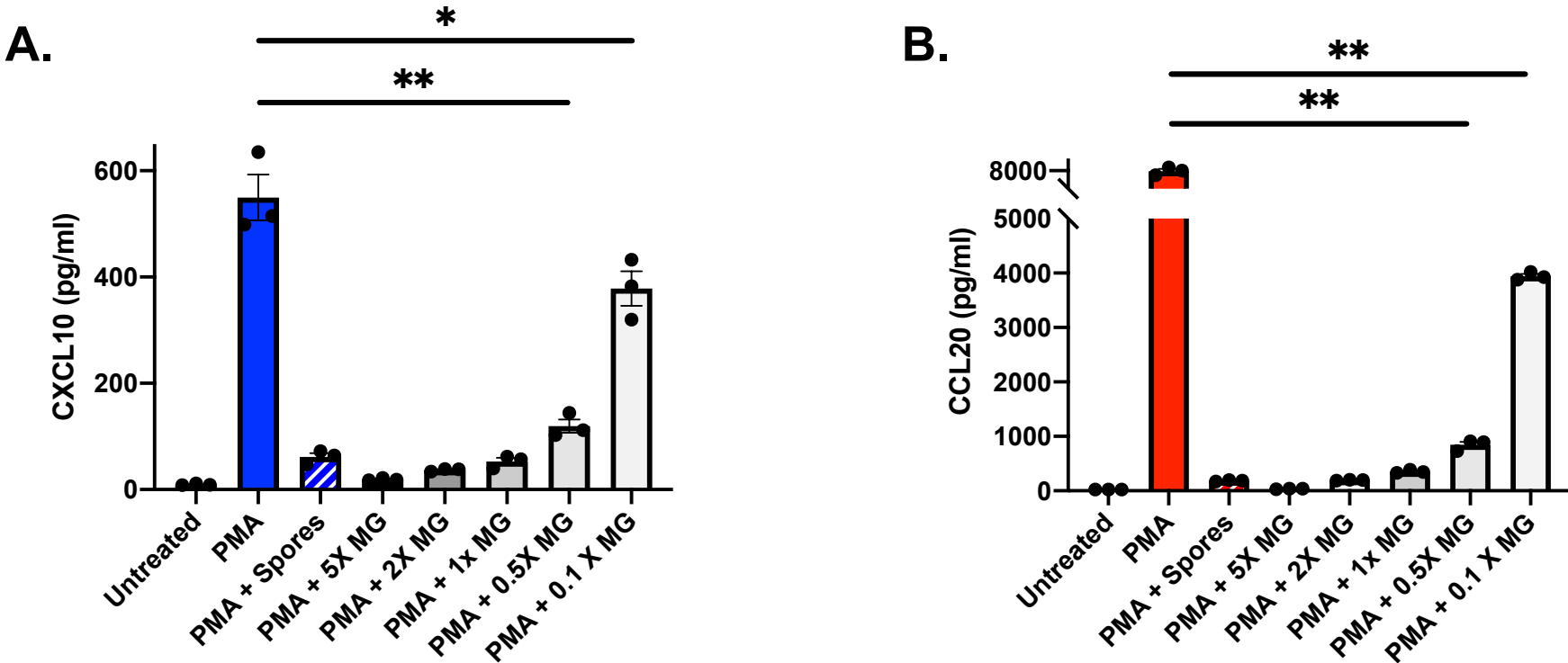

Supplement: FIG S6 [file mbio.00194-23-s0007.pdf]
