# Supplementary material for: Depletion of Extracellular Chemokines by Aspergillus Melanin
Source: mBio. 2023 Apr 17;14(3):e00194-23. doi: 10.1128/mbio.00194-23 (PMC10294650; doi:10.1128/mbio.00194-23)
Supplement: TEXT S1 [file mbio.00194-23-s0001.docx]

**Supplemental Information**

**MATERIALS AND METHODS**

**Fungal strains and human cell line.**

All *Aspergillus fumigatus* strains, including Af293 ([1](#_ENREF_1)), CEA10 ([2](#_ENREF_2)), B5233 ([3](#_ENREF_3)), B5233/RGD12-8 (pksP∆ mutant) ([3](#_ENREF_3)) and RGD12-8-PKS33-3 (pksP∆ complemented strain) ([3](#_ENREF_3)), were grown on peptone-dextrose agar (PDA) plates for 3 to 5 days at 37°C prior to use. Spores were collected in endotoxin-free Dulbecco’s phosphate-buffered saline (DPBS), washed with endotoxin-free DPBS, and counted with a hemocytometer to prepare the final inocula. HSAEC1-KT cells (CRL-4050) were purchased from ATCC were maintained in flasks with SAGM (Small Airway Epithelial Cell Growth Medium Basal Medium; Lonza) at 37 °C, 5% CO2. The cells were fed every 2 – 3 days and passaged once a week. The protease inhibitor cocktail was purchased from Sigma-Aldrich (P1860) and was diluted 1:200 in the SAGM media when indicated. Recombinant CXCL10 and CCL20 was purchased from R&D Systems.

**In vitro infection**

Infections were performed in 6-well plates. 6 days prior to infection, 2.5 x 10^5^ cells were seeded into each well in 2 ml media and allowed to reach 80 - 90% confluence. The HSAEC1-KT cells were infected with 8.6 × 10^6^ spores suspended in 1.5 ml of SAGM medium, approximating a multiplicity of infection (MOI) of 5 spores per host cell. Prior to infection, the spores were incubated in SAGM medium @ 37°C for 1 hour to initiate conidial swelling. At the indicated time post infection, supernatants were collected and immediately frozen and -80°C and cells were resuspended in 1 ml of Trizol reagent and frozen at -80°C for subsequent RNA isolation. For experiments where PMA was used to induce chemokine gene expression, HSAEC1-KT cells were with pretreated with PMA (0.1µM) for 1 hr prior to addition of spores as well as for 6 hours following the addition of spores. Where indicated, spores were heat-killed by incubation @ 65°C for 2 hours and verified by plating an aliquot onto a PDA plate for 2 days at 37°C after which no fungal growth was observed. When melanin ghosts were added to the host cells, they were counted using a hemacytometer prior to addition for an MOI of approximately 3 melanin ghosts per host cell.

**Cytokine analysis**

Cytokines were measured by two-antibody ELISA using biotin-streptavidin-peroxidase detection. Polystyrene plates (Maxisorb; Nunc) were coated with capture antibody in PBS overnight at 25°C. The plates were washed 4 times with 50 mM Tris, 0.2% Tween-20, pH 7.0-7.5 and then blocked for 90 minutes at 25°C with assay buffer (PBS containing 4% BSA (Sigma)). Then 50µl of sample or standard prepared in assay buffer was incubated at 37°C for 2h. The plates were washed 4 times and 100µl of biotinylated detecting antibody, in assay buffer, was added and incubated for 1h at 25°C. After washing the plate 4 times, streptavidin-peroxidase polymer in casein buffer (RDI) was added and incubated at 25°C for 30min. The plate was washed 4 times and 100µl of commercially prepared substrate (TMB; Dako) was added and incubated at 25°C for approximately 10-30 min. The reaction was stopped with 100µl 2N HCl and the A450 (minus A650) was read on a microplate reader (Molecular Dynamics). A curve was fit to the standards using a computer program (SoftPro; Molecular Dynamics) and cytokine concentration in each sample was calculated from the standard curve equation.

**qRT-PCR**

Total RNA was isolated from HSAEC1-KT cells using the PureLink RNA isolation kit (Invitrogen #12183020). 500 ng total RNA was reverse transcribed using Maxima H Minus cDNA Synthesis Master Mix (M1681, Thermo Scientific). Quantitative PCR (qPCR) was performed using KAPA SYBR FAST qPCR Master Mix Kit (KK4601, Kapa Biosystems) and 10 µM each of forward and reverse transcript-specific primers (Table 1), using a CFX 384 real time system (Biorad). The program consisted of one cycle of 95 °C for 3 min, 40 cycles of 95 °C for 3-sec, 60 °C for 40 sec (annealing and polymerization) followed by melting curve analysis. Data were analyzed using CFX Maestro software (Bio-Rad). Relative mRNA levels for genes were normalized to the geometric mean of the endogenous controls GAPDH, and RPL13A using the 2^-ΔΔCt method. Analysis of relative gene expression data using real-time quantitative PCR and the 2− ΔΔCT method ([4](#_ENREF_4)).

**Preparation of melanin ghosts**

To isolate melanin ghosts, we followed the protocol provided by Dr. Radames JB Cordero (US patent number [US20220042056A1](https://nam11.safelinks.protection.outlook.com/?url=https%3A%2F%2Fpatents.google.com%2Fpatent%2FUS20220042056A1%2Fen%3Finventor%3Dcordero%2Bradames%26oq%3Dcordero%2Bradames&data=05%7C01%7Cvbruno%40som.umaryland.edu%7C7e523f431dd744c36dbd08dafa89c6fd%7C717009a620de461a88940312a395cac9%7C0%7C0%7C638097767260789729%7CUnknown%7CTWFpbGZsb3d8eyJWIjoiMC4wLjAwMDAiLCJQIjoiV2luMzIiLCJBTiI6Ik1haWwiLCJXVCI6Mn0%3D%7C3000%7C%7C%7C&sdata=2YpoHFU7tGEFHoO5slaNgoPXfQQVKkYGi9rtsEIrifA%3D&reserved=0)). Briefly, melanized *A. fumigatus* conidia were collected by centrifugation at 4,000 rpm for 20 minutes. An equal volume of 6N HCL was added to each pellet followed by incubation at 100 ℃ for 1 hr followed by centrifugation at 4,000 rpm for 15 minutes. The supernatant was decanted and the pellets were washed with PBS 3 times. Three consecutive Folch lipid extractions were performed using a 8:4:3 chloroform/methanol/water ratio. Pellets were washed 3 times with water and resuspended in water. The melanin ghosts were enumerated with a hemacytometer prior to adding to PMA-treated host cells.

**References**

1. Nierman WC, Pain A, Anderson MJ, Wortman JR, Kim HS, Arroyo J, Berriman M, Abe K, Archer DB, Bermejo C, Bennett J, Bowyer P, Chen D, Collins M, Coulsen R, Davies R, Dyer PS, Farman M, Fedorova N, Fedorova N, Feldblyum TV, Fischer R, Fosker N, Fraser A, Garcia JL, Garcia MJ, Goble A, Goldman GH, Gomi K, Griffith-Jones S, Gwilliam R, Haas B, Haas H, Harris D, Horiuchi H, Huang J, Humphray S, Jimenez J, Keller N, Khouri H, Kitamoto K, Kobayashi T, Konzack S, Kulkarni R, Kumagai T, Lafon A, Latge JP, Li W, Lord A, Lu C, et al. 2005. Genomic sequence of the pathogenic and allergenic filamentous fungus Aspergillus fumigatus. Nature 438:1151-6.

2. Girardin H, Latge JP, Srikantha T, Morrow B, Soll DR. 1993. Development of DNA probes for fingerprinting Aspergillus fumigatus. J Clin Microbiol 31:1547-54.

3. Tsai HF, Chang YC, Washburn RG, Wheeler MH, Kwon-Chung KJ. 1998. The developmentally regulated alb1 gene of Aspergillus fumigatus: its role in modulation of conidial morphology and virulence. J Bacteriol 180:3031-8.

4. Livak KJ, Schmittgen TD. 2001. Analysis of relative gene expression data using real-time quantitative PCR and the 2(-Delta Delta C(T)) Method. Methods 25:402-8.
